# Special Characteristics of the Resilience of Russian Families in the Face of Modern Challenges (A Preliminary Study)

**DOI:** 10.11621/pir.2022.0304

**Published:** 2022-09-30

**Authors:** Maria A. Odintsova, Dmitry V. Lubovsky, Polina A. Ivanova, Elena S. Gusarova

**Affiliations:** a Moscow State University of Psychology & Education, Moscow, Russia

**Keywords:** loss challenges, relationship challenges, global challenges, challenges of illness, challenges of financial well-being, family resilience

## Abstract

**Background:**

A challenge is considered a “wake-up call” for family resilience, requiring a proper response (willingness to evaluate, understand an event and its signals, and also give an adequate response). Family resilience is defined as an adequate response to challenges, that is, the ability to cope with them based on the cultural-historical context and family resources, such as clear and open family communication and connectedness, the use of social resources, a broad system of values and senses, the ability to derive meaning from adversity, acceptance, and flexibility.

**Objective:**

This article reports on a study which aimed to analyze components of the resilience of Russian families in response to life circumstances that have become challenges for them.

**Design:**

The study was conducted from March 20 to May 7, 2022. Participants took an online survey on Yandex-forms; the link to the survey was distributed through social networks on public pages and in private messages. Two hundred seventy-four (274) representatives of Russian families responded, including 234 women and 40 men (14.6%) ranging from age 17 to 65 (cf. 34.1+12.5). After removing the data of 15 participants who did not report a negative event in their families, the final sample consisted of 259 people.

**Results:**

Challenges for modern Russian families can be categorized as loss challenges, relationship challenges, global challenges, challenges of illness, and challenges of financial well-being. The challenges of loss stimulate flexibility of response, acceptance, and overcoming suffering through mutual understanding. Global challenges and the challenges of illness awaken family spirituality. The ability to discuss problems together and share decision making becomes a resource to meet the challenge of families’ financial well-being. Confidence in solving problems and a positive outlook become resources to face relationship challenges. The intensity of events is a signal for a family to evoke communication and connectedness, acceptance, and flexibility, as well as family resilience as a whole.

**Conclusion:**

There is a connection between the difficulties that respondents regard as challenges and the characteristics of their family resilience; the most adequate response to challenges is to increase family resilience.

## Introduction

The challenges that families are facing today are becoming more intense and global in nature. They require conceptualizing the category of challenge itself and the situations associated with it. Back in 1935, A. Toynbee formulated the law of challenge and response in his book *A Study of History.* His chapter entitled “Challenge and response” asserted that this law determines the development of history and society.

Toynbee highlighted the following external types of challenges: nature, history, and religion; strike (war); pressure and punishment (discrimination); a new environment (the challenge of change) (Toynbee, 1934/2001); time (Vodyakha et al., 2021); uncertainty; achievements; threats (Leontiev, 2011); and the challenges of everyday life (Odintsova, 2019). The internal ones Toynbee identified were as follows: existential (Grishina, 2011); self-design (personal reflection, creative identity, cognitive-personal style, belief in the ability to cope with a creative task, facilitated communication) (Vodyakha et al., 2021); adaptation, pre-adaptation, and multiple identities against the backdrop of uncertainty; complexity; and diversity (Asmolov, 2015).

The family itself also faces challenges to its structure and reality (Minukhin, & Fishman, 1998; Dobrokhleb, & Ballaeva, 2018) as follows: challenges in the context of illness/disability (Vikström et al., 2020; Chew, et al., 2018; Fitryasari, 2018) and the role of raising children with disabilities in such families (Qiu et al., 2021); the challenge of cultural identity in building family resilience (Pudjiati et al., 2021); the challenge of family identity that reinforces the sense of belonging to a group (“we are a family”) and protects the well-being of its members (Muldoon et al., 2019); migration (Valade, 2021); poverty (Silva et al., 2021; Erdem et al., 2021); natural disasters (Sadia et al., 2020); trauma and loss in the pandemic (Walsh, 2020); illness and death of close relatives; family relationships’ challenges, and financial difficulties (Gonzalez-Mendez et al., 2022).

In a broad sense, a challenge is a trial in the context of a sharp deterioration of society’s living conditions, a problem that people must solve in order to survive and develop (Toynbee, 2001). This is a demand that “is made by some external (in relation to our Self) situation” (Leontiev, 2011, p. 450), a demand calling for one or another kind of active response. N.G. Grishina defines challenges as existential problems related to age, significant changes in life situations, and an increasing need for internal changes that requires a constant response at different stages of life (Grishina, 2011).

Based on the linguistic interpretation of the word “challenge,” M. Tashlykova summarizes: “the noun ‘challenge’ is increasingly detached from the description of the initial situation of interpersonal interaction and acquires the ability to be used to characterize such a situation, which is generated by interaction of other forces” (Tashlykova, 2015, p. 92).

Thus, a challenge is a message, a test, a problem, a contradiction, or psychological task that includes different situations and events as well as requirements to grow in response to this task, and to properly respond at the right time (willingness to assess, perceive, understand the situation and its signals, and also to give an adequate response). Obviously, when a person himself cannot provide an adequate response to challenges of our time, the response can be made by a microsocial group to which he belongs. After all, “the ability of an individual to adapt to challenges depends on their connections with other people and systems external to the individual through relationships and other processes” (Masten, & Barnes, 2018).

One such group is a family as a community of similarities (Kaufman, 2007; Prien, 2004). A family as a living system (Bowen, 1978; Chernikov, 2001; Sameroff , Mackenzie, 2003), united by common feelings, goals, consonance of views, mutual responsibility, the need to feel connected with others, and, finally, common history and culture, can “endure the most severe forms of suffering and loss, and with time and joint efforts recover and become stronger,” and more resilient (Walsh, 2020, p. 910).

Adaptability is considered one of the most important properties that ensure the normal functioning of the family as a system. Maladaptation as a mark of dysfunction, expressed in inadequate responses to challenges, can lead to decline and degradation of the family as a microsocial system (Chernikov, 2001). Even though cultural patterns are changing more slowly in family life than in society, general changes in the family sphere are becoming more noticeable today. The instability of family structure (Kovalyova, 2021), more frequent divorces (Kharitonova, 2021), the increasing optionality of marriage (Kobleva, 2021), the decreasing birth rate (Vartanova, 2021), etc. are also responses to the challenges of our time, although not adequate. They are caused by “unexpected disturbances and/or… disruptive changes, and/or… chronic mismatch between life and expectations” (Evans, 2019); impoverishment of the family environment (unfavorable family climate, defects in raising a child, blurring of boundaries, etc.) (Bystrova, & Khizhnaya, 2018); family disorders (functional, structural, role-playing, communicative, systemic) (Nikolskaya, 2010); and dysfunctions in parental families (parental criticism, anxiety induction, elimination of emotions and fixation on negative emotions, etc.) (Kholmogorova et al., 2016).

Thus, a family is sensitive to a system of external and internal conditions for its functioning that affect how family members perceive, understand, and transform specific situations and challenges; how they conceptualize them as a whole; and how they search and find opportunities to mitigate and overcome the impact of challenges throughout family life (Walsh, 2020). A family develops in an interconnected set of different external conditions (cultural and historical conditions, situations, events, environments) and internal prerequisites (family functionality, readiness to act and overcome, resilience, etc.). The diversity of internal prerequisites contributes to adequate family responses to challenges; poverty contributes to inadequate responses. But even the absence of challenges leads to stagnation and slow degradation.

At the same time, the family’s potential for adaptation to one situation is transferred to others, thereby allowing families to show resilience in a new environment, that in its turn can become a new challenge. Otherwise, developmentally inappropriate, insufficiently supportive, and culturally incongruent environments become threatening, exacerbate stress, and hinder the development of family resilience (Walsh, 2020). E.V. Kuftiak enlarged this idea: “Life difficulties experienced by a family can be metaphorically compared to a wake-up call, focusing on what is important. A critical event encourages family members to pay more attention to relationships, values, and life goals” (Kuftyak, 2014).

As one can see, the main answer to modern challenges is family resilience, which can be defined as

- the path that the family follows, adapting to difficulties and rising to overcome them; positively responding to challenges in unique ways, depending on context, developmental level, an interactive mix of risk and protection factors, as well as the common position of family members (Valade, 2021);
- the ability to reduce the impact of stressful situations through relationships that allow the impact of demands, helping people to find resources, and to expand or to create them over time;
- the ability to successfully overcome difficulties that threaten its functioning and development as a system (Southwick, 2014); and
- a “dynamic systemic characteristic of a family able to respond to stresses of various origins using its own protective factors and family resources (individual, of family as a system, environmental), suggesting that a family is able and willing to cope, change, adapt, and develop” (Makhnach, & Laktionova, 2021).

All these definitions emphasize the dynamism of family resilience: it is a process that cannot be considered separately from some event or situation. However, the definitions of family resilience do not always take into account cultural and historical aspects, although it is recognized that a resilient family, better than other institutions of society, can provide stable reproduction of culture and values, emotional and spiritual communication, connectedness, security, safety, and resourcefulness. Family resilience has been proven to be related to individual resilience (Bourbeau, 2018) and to the resilience of society and culture (Zuev, 2018). Culture is one of the key factors in shaping family resilience, since the cultural and historical context of society shape how people understand the value of family resilience.

Historical events of the past (Linchenko, 2020), and values, traditions, and beliefs (Artsiomenka, 2019), conditioned by the cultural and historical context, can become powerful means of resisting adversity. This foundation allows families to solve problems of cognitive dissonance (clashes between conflicting ideas, values, and beliefs); overcome crises that families inevitably face at different stages of their functioning; accept events that make them vulnerable if it is impossible to change circumstances; and adapt to them, becoming more flexible or restructuring their views and goals so that they are compatible with those that exist in society.

At the same time, families’ rigid attitudes towards the cultural and historical context of the past can become an obstacle to meeting modern challenges. Family resilience leads to an adequate response to challenges and provides the ability to cope with them based on the cultural and historical context and family resources. Thus, family resilience can be seen as the ability to cope with both everyday and exceptional challenges based on the cultural-historical context and family resources: clear and open family communication and connectedness; the use of family social resources; the ability to unite and use all their resources when faced with an adverse event; sharing a broad system of values and meanings; ability to make meaning of adversity; and acceptance and flexibility.

Research on the modern challenges and factors of family resilience in the face of these challenges has revealed a wide range of both internal and external factors determining family resilience. Meanwhile, there has been insufficient study of how special characteristics of family resilience relate to various types of challenges.

Research has also shown that family resilience is not absolute: in relation to some challenges, a family can show high resilience, while another challenge can turn into a family disaster. Coping with some challenges becomes an opportunity for the family to grow, while coping with others becomes hard and fruitless work. In this regard, it is especially important to study what life difficulties families regard as challenges, since the world is rapidly changing under conditions of transition and, accordingly, the subjective picture of life difficulties as challenges to family resilience is changing in the minds of family members.

So, what resources of resilience do Russian families have, depending on the specifics of the life circumstances that become challenges for them? This problem is especially important in the context of providing psychological assistance to families, since the content of the challenges for which families seek help can be a heuristic for exploring their resilience resources and further work on their development.

### Objective

This study aimed to analyze components of the resilience of Russian families as responses to life circumstances that become challenges for them.

Our research goals were:

1. to identify events and difficult life situations that representatives of Russian families see as challenges;
2. to assess the level and intensity of the family’s resilience components; and
3. to analyze the correlation between the challenges faced by Russian families and the components of family resilience mobilized as responses to these challenges.

Our hypotheses were that family members united by similar adverse events that have become challenges for them will evaluate the intensity of experienced events and changes in family relationships differently than those experiencing different types of adverse events, as well as utilize different components of family resilience.

## Methods

### Participants

Anyone could take part in this study, and from the start, potential respondents were informed about our goals and objectives. The study was conducted from March 20 to May 7, 2022. Respondents took the online survey on Yandex-forms; the relevant link was distributed through public pages and private messages in social networks. The study recruited 274 Russian people, including 234 women and 40 men (14.6%) ranging in age from 17 to 65 (cf. 34.1+12.5).

The sample was not balanced by gender, which is due to the greater willingness of women to participate in such surveys. In answering the questions, the respondents characterized their families as microsocial systems. Biographical information about the respondents is presented in *Table 1.*

**Table 1:** Biographical data of the study participants

Education:	Secondary (high school)	Secondary specialized	Higher	
	55 (20.1%)	46 (16.8%)	173 (63.1%)	
Occupation:	Working	Studying	Both working and studying	Other (maternity leave or retired)
	131 (47.8%)	97 (35.4%)	16 (5.8%)	30 (10.9%)
Marital status:	Single	Married	In relationship or in unregistered marriage	Other (divorced, widow/er)
	94 (34.3%)	116 (42.3%)	47 (17.2%)	17 (6.2%)
Disability	Parents of a child with disability	Parents	Number of children 59 parents (21.5%) have 1 child; 56 (20.4%) – 2; 21 (7.7%) – 3;	
43 (15,7%)	23 (8.4%)	146 (53.3%)	3 (1.1%) – 4; 1 (0.4%)– 6.	

### Questionnaires

1. Biographical questionnaire (sex, age, education, occupation, disability, marital status, children, including children with disabilities, number of children).
2. Family Resilience Assessment Scale (Gusarova et al., 2021). Before evaluating statements about their family experience, the respondents were asked to indicate one of adverse events in their family and the level of intensity of this event for the family, and answer the question: “After this event, did you feel that the relationship in your family (choose the answer that is most typical for your family): 1) became more distant (you moved away from each other); 2) remained the same; or 3) became closer (you became closer to each other).” These questions pertained to the respondents’ families (for those who have created a family) or to parental families (for single people).

Data processing included descriptive statistics, χ^2^test, the non-parametric Kruskal–Wallis test to assess the significance of differences between groups, Cronbach’s α to assess the internal consistency of the Family Resilience Scales, and correlation analysis (Spearman’s ρ).

## Results

Many participants reported having experienced at least one major negative experience in their family life. Fifteen people (5.5%) wrote that there were no adverse events in their families, including six men. This group turned out to be the “youngest” (average age 24.5+8.1) who marked non-existent events with one point of intensity and was therefore removed from the subsequent statistical analysis. Thus, the final sample consisted of 259 people. The events that the respondents identified as challenges were sorted into five groups. (See *Table 2*) In order of frequency, they were:

1. Death of close relatives (parents, grandparents, husband, wife, brothers, sisters) — Loss challenges (24.7%).
2. Negative family relationships (conflicts, quarrels, scandals, divorce, etc.) — Relationship challenges (23.6%).
3. Many problems in the family at the same time (moves, loss of relatives; divorce, job loss, birth of a child with a disability; divorce and death of close relatives; military special operation in Ukraine, participation of a spouse in hostilities; financial difficulties, death of a child, etc.) — Global challenges (22.8%).
4. Illnesses of close relatives, including severe diseases of children — Challenges of illness (5.1%).
5. Economic difficulties (lack of funds; job loss; home loss, etc.) — Challenge of financial well-being (13.9%).

**Table 2:** Average age and average estimates of the events intensity among representatives of groups united by similar events/challenges

Challenges			Average group age		Intensity of events	
	N	%	M	SD	M	SD
Loss	64	24.7	33.2	13.1	8.6	2.2
Relationship	61	23.6	30.9	12.7	7.5	2.7
Global	59	22.8	37.7	11.7	8.9	1.8
Illness	39	15.1	38.0	11.4	8.6	2.0
Financial well-being	36	13.9	34.8	11.7	7.2	3.3
Total	259	100	34.1	12.5	8.2	2.5

People in these different groups differed in age (F = 3.263; p = .012) and in estimates of the intensity level of the adverse events (F = 4.837; p = .001) in their families. Those who noted many adverse events in the family (global challenges) and events associated with severe diseases in families (challenges of illness) were on average about 40 years old; those who experienced loss (death of close relatives), as well as relationship challenges, on average just over 30 years old; those who indicated events associated with serious financial difficulties were about 35 years of age. All events were rated quite high in terms of their intensity: on average, from 7.2 to 8.9 points out of a possible 10. However, global challenges (8.9 points), loss challenges (8.6 points), and challenges of illness (8.6 points) scored the highest.

There were no statistically significant differences in types of challenges between the group with children and the childless one (χ^2^= 8.763; p = .067); between families with and without disabilities (χ^2^= 5.3373; р = .254); or between groups with different employment statuses (χ^2^= 15.460; p = .217). At the same time, there were statistically significant differences in the types of events/challenges between groups with different marital status (χ^2^= 31.586; p = .002). Challenges of illness were marked more by married respondents; relationship challenges by unmarried/single respondents and respondents in unregistered marriages; global challenges and loss challenges by widows/widowers and divorced respondents (group “Other”). (See *Table 3*)

**Table 3:** Types of challenges in groups of respondents with different marital status

	Illness	Loss	Relationship	Economic difficulties	challenges Global
Unmarried/single	10.1%	24.7%	33.7%	12.4%	19.1%
Married	22.0%	24.8%	11.0%	19.3%	22.9%
In a relationship	13.6%	22.7%	34.1%	9.1%	20.5%
Other	0.0%	29.4%	23.5%	0.0%	47.1%

Next, we analyzed the respondents’ answers to the question: “After this event, did you feel that the relationship in your family (choose the answer that is most typical for your family): 1) became more distant (you moved away from each other); 2) remained the same; of 3) became closer (you became closer to each other).

**Figure 1.**
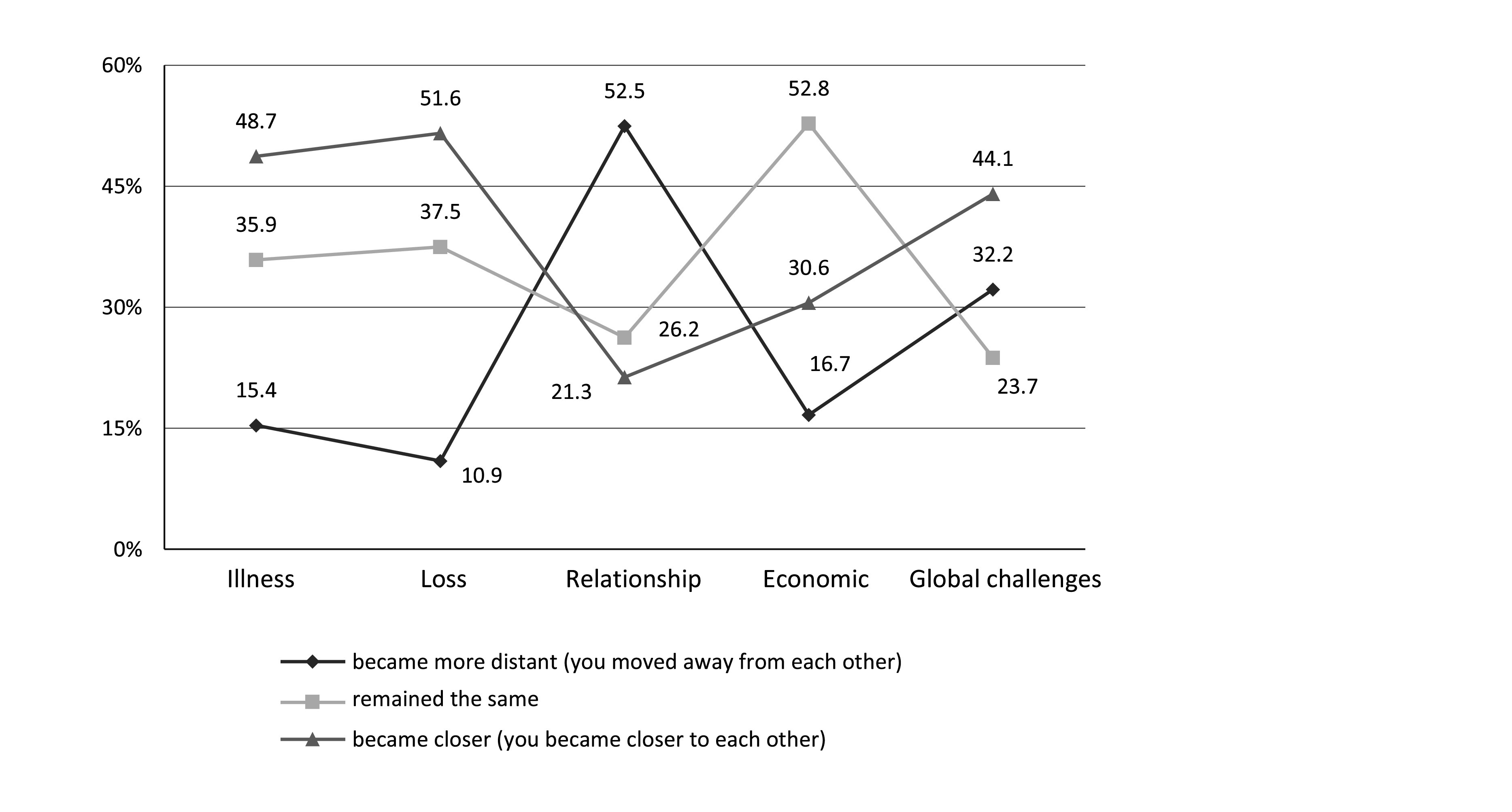
Changes in family relationship after adversity (% of respondents)

The analysis showed that different types of events influenced family relationships in different ways (χ^2^= 41.063; р = .000). Relationship challenges made connections with the family more distant (people moved away from each other); in case of challenges of financial well-being, they often remained the same; challenges of illness, loss, and global challenges made relationships in family closer. (See *Figure 1*)

Next, we analyzed differences in the characteristics of family resilience under different types of challenges. Initially, the internal consistency of the family resilience assessment scale and its subscales were checked with the Cronbach’s α coefficient. Previously, this scale had been adapted by us, and adequate psychometric properties had been identified. However, in this study, the response range was expanded to five options (Always = 5 points; Often = 4 points; Sometimes = 3 points; Rarely = 2 points; Never = 1 point).

It is generally accepted that the 4-point scale does not allow respondents to express a neutral answer; therefore it is called the “forced” scale (forced Likert scale) and is more often used in marketing research to force respondents to make up their minds. This approach results in more questions going unanswered. Some researchers (Krosnick, 1991; Østerås, 2008) also consider scales with an odd number of alternative answers to be the most optimal. On the one hand, the larger the scale in terms of range, the higher its reliability, validity, and discrimination; on the other hand, wider scales in terms of range are more difficult for respondents (Preston and Colman, 2000; Taherdoost, 2019).

Therefore, we struck a balance between informativeness and ease of filling out the questionnaire. In addition, the feedback we received from respondents when testing the psychometric characteristics of the questionnaire showed the limitations of the 4-point scale. The range from 1 to 5 turned out to be the most acceptable, as it included the intermediate answer “Sometimes.” A family spirituality scale was also added. In the previous version (Gusarova, 2021), this scale did not pass the test, since in the original version it was focused mainly on a narrow (religious) understanding of spirituality, and not spirituality in the understanding of F. Walsh. Other researchers have also noticed this (Chiu, 2019; Chow, 2022; Gardiner, 2019).

Following aforementioned authors’ recommendations, and especially those of E. Gardiner et al., we formulated the following seven statements: 1) “We focus on common family moral principles;” 2) “We support common family traditions;” 3) “We create new family traditions;” 4) “Art, music and literature help our family to cope with difficulties and stress;” 5) “The experience we had has made us able to sympathize with others;” 6) “Common meanings and values help us to overcome difficulties;” and 7) “To cope with adversity, we rely on the spiritual resources of our family.” The Cronbach’s α coefficient for each subscale indicated a high level of consistency: family communication and connectedness — α = 0.966; positive outlook and problem solving — α = 0.937; acceptance and flexibility — α = 0.891; family social resources — α = 0.913; and family spirituality – α = 0.921). The Cronbach’s alpha for the summative scale was 0.976, which is very high.

**Figure 2.**
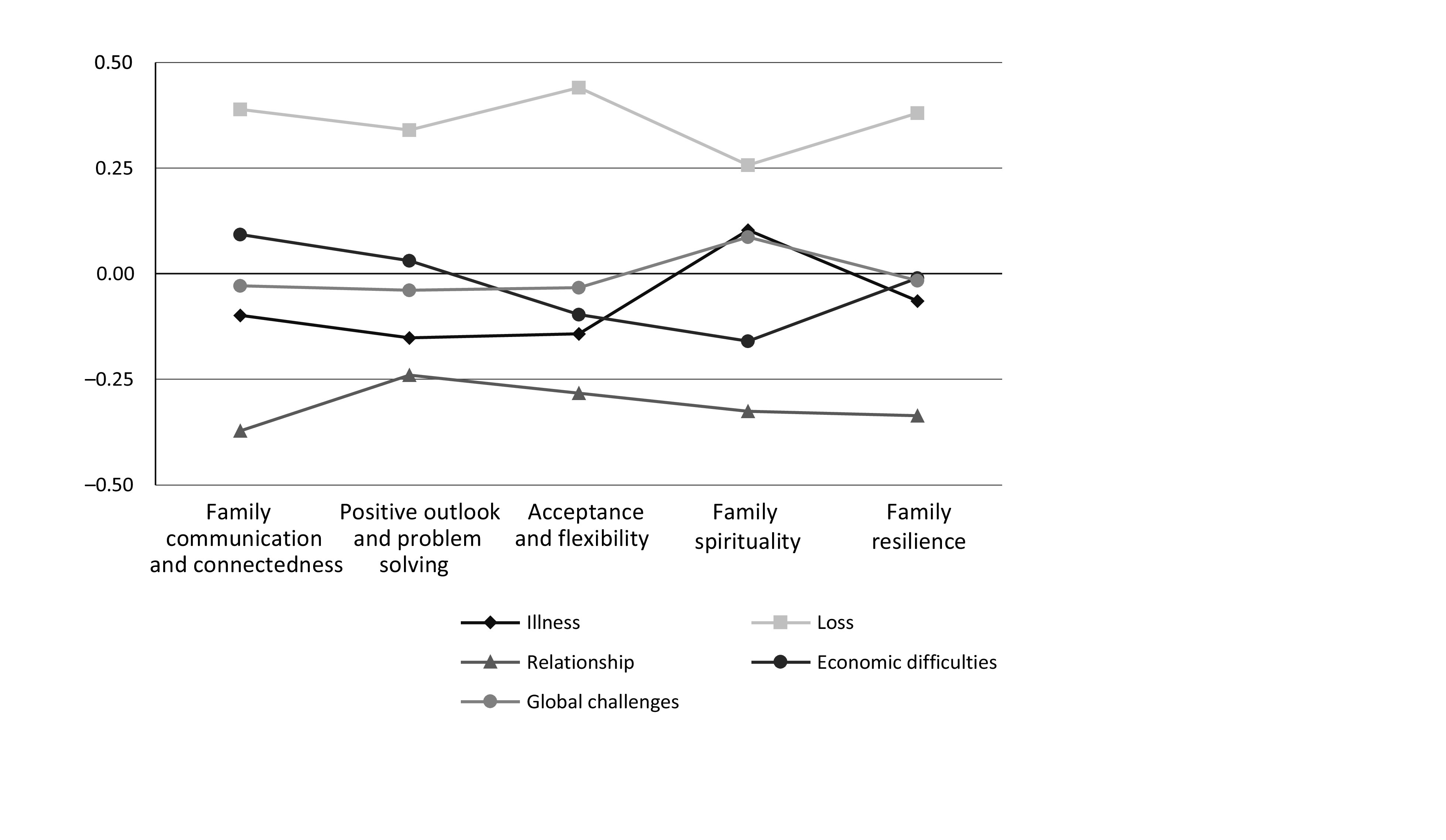
Family resilience by challenge types (normalized data)

The Kruskal-Wallace test was used to detect differences in the components of family resilience for different types of challenges, since the distribution on some scales differed from the normal one. It was found that people grouped according to different types of events/challenges differed in the following characteristics of family resilience: family communication and connectedness (H = 15.242; p = 0.004); acceptance and flexibility (H = 22.050; p = 0.000); family spirituality (H = 10.638; p = 0.031) and family resilience as a whole (H = 11.338; p = 0.023). (See *Table 4, Figure 2.*)

**Table 4:** Average rates of family resilience among representatives of groups united by various challenges

	Challenges											
	Illness		Loss		Relations- hip		Economic difficulties		Global challenges			
	M	SD	M	SD	M	SD	M	SD	M	SD	Н	Р
Family communication and connectedness	56.9	11.4	63.1	9.2	53.5	15.1	59.4	12.2	57.8	12.2	15.24	.004
Positive outlook and problem solving	30.2	5.9	33.2	4.8	29.7	7.4	31.3	5.8	30.9	5.4	8.87	.064
Acceptance and flexibility	18.7	3.5	20.9	3.3	18.2	4.1	18.9	4.4	18.7	3.0	22.05	.000
Family spirituality	25.9	6.1	26.8	4.9	23.3	6.9	24.3	5.4	25.8	5.5	10.64	.031
Family resilience	153.9	27.8	166.9	22.5	145.9	36.5	155.5	27.2	155.3	26.5	11.34	.023

*Table 4* presents the mean values and standard deviations for all characteristics of family resilience. The data was preliminarily normalized through z-values. *Figure 2* reflects how the studied characteristics determined the key resources of the resilience of each family group. Data standardization allowed us to eliminate the possible influence of deviations in properties and to highlight the dominant characteristics in the family resilience profile of each group.

All components of family resilience were most highly rated by the group united by the challenge of loss (death) of close relatives. This group was especially flexible in responding to unforeseen circumstances, recognizing the unexpectedness of events, accepting them as part of life, and overcoming suffering through mutual understanding. Family spirituality (common values, meanings, moral principles, life experience) was the dominant resource for two groups: the one united by global challenges as adverse events, and the other by challenges of illness. The group that indicated events related to economic problems (lack of funds, job loss, home loss, etc.) expressed the ability to discuss problems together and to share decision making in the family, but the indicators of family spirituality were lower. The group united by relationship challenges as adverse events (conflicts, quarrels, scandals, divorces, etc.) turned out to be the most vulnerable. However, their confidence about solving problems and positive outlook stand out in contrast to other lower components of family resilience.

The relationship between the subscales of family resilience and age, number of children, intensity of events, and changes in family relationships were analyzed. The correlation analysis revealed significant results: family spiritual resources increased with age (ρ = .253; p < .01) and correlated with family connectedness (ρ = .353; p < .01). Families with many children showed higher scores of family spirituality (ρ = .287; p < .01). Such families had more resources of family communication and connectedness (ρ = .161; p < .01), a positive outlook toward solving problems (ρ = .205; p < .01), and higher resilience scores (ρ=.188; p < .01). The higher the intensity of an adverse event, the closer family communication and connectedness became (ρ = .176; p < .01). The family accepted the event and reacted flexibly to it (ρ = .192; p < .01); in general, family resilience increased (ρ = .153; p < .05). Finally, going through adverse events made family relationships closer, which, in turn, enhanced all the resources of family resilience: family communication and connectedness (ρ = .385; p < .01); positive outlook and problem solving (ρ = .324; p < .01); acceptance and flexibility (ρ = .341; p < .01); family social resources (ρ = .182; p < .01); family spirituality (ρ = .353; p < .01); and the general level of family resilience (ρ = .384; p < .01). The results of the correlation analysis are presented in *Table 5.*

**Table 5:** Relationship between components of family resilience, age of respondents, number of children, intensity of events and family relationship (Spearman’s rho)

Components of resilience	Age	Number of children	Intensity of events	Family relationships
Family resilience	.131*	.188**	.153*	.384**
Family edness communication and connect-	.109	.161**	.176**	.385**
Positive outlook and problem solving	.149*	.205**	.101	.324**
Acceptance and flexibility	.030	.084	.192**	.341**
Social resources	.045	.093	.053	.182**
Family spirituality	.253	.287**	.106	.353**

Note: ** = p < .01, * = p < .05

The Family Resilience Assessment Scale (Gusarova et al., 2021) has one open question: “Is there anything else that was not described or discussed above that helped your family to overcome the adverse event?” This question was not mandatory; however, 29 people answered it. The most frequent answers were the following:

1. “We are together” (unity; joint activities and cares; mutual assistance, valuing each other; common traditions and family values passed down from generation to generation; respect for each other; our connection) (N = 11);
2. Personal qualities and feelings (tolerance; willingness to change lifestyle and social circle; maturity and disillusionment of youth; faith, hope, and love; trust, empathy, understanding, and self-confidence) (N = 10);
3. Philosophical view of the world and life (faith in God; time, knowledge) (N = 5);
4. Self-sacrifice (avoiding an open discussion of a negative event; unwillingness to remember a negative event, hiding the truth from family members to preserve their well-being) (N = 3).

## Discussion

Our study is one of the few in which the characteristics of the resilience of Russian families are considered in relation to the events and difficulties that became challenges for them. The legitimacy of combining the research participants into groups according to the events that they identified as challenges was confirmed by the following positions.

1. Loss challenges, relationship challenges, global challenges, challenges of illness, challenges of financial well-being – these are what our respondents wrote about, and they are the foci of most research on family resilience (most often each challenge is considered separately). Thus, special characteristics of family resilience have been analyzed in relation to illness in the family (Saltzman, 2016); to loss (Barboza, 2021; Khatib, 2022); conflicts in the family and divorce (Mashego, 2014; Strizzi, 2022); financial difficulties (Kholostova, 2018; Orthner, 2004; Raniga, 2016); to global challenges, including various problems, for example, military operations, natural disaster, migration (Denoy, 2019; Mawarpury, 2017); and low income, together with potentially fatal disease (Naidoo, 2022).
2. The generalized analysis of family challenges presented in the work of M. Lin et al. (Lin et al., 2016) partially corresponds to what we have done. Among the challenges families face, the M. Lin et al. also highlighted: 1) developmental problems of children and 2) family members’ hesitation to share family duties. They also distinguished: 1) familial financial problems and breadwinner’s unemployment, and 2) unexpected medical crises of family members and chronic illness of children. Since our study was preliminary and the sample was small, we highlighted more generalized groups of challenges.
3. Global challenges, loss challenges, and challenges of illness were rated with the highest estimates of the intensity of events experienced by our study’s participants. The potentially high intensity and stressfulness of such events in family life produces negative experiences and a decrease in the quality of life of widows and widowers (Hasida Ben-Zur, 2012), and the quality of life and the severity of post-traumatic symptoms in family members of chronically ill patients (Wintermann, 2016).
4. It is quite natural that unmarried/single respondents and those who are in an unregistered marriage more often spoke about relationship challenges. They perceived the difficulties that create risks for relationships with loved ones as a challenge. The more frequent mention of this challenge by these groups is due to:
  - The instability of their marital status. After all, an unregistered marriage, being an intermediate form of marital status between legal marriage and loneliness, absorbs all the difficulties of a marriage union, and exacerbates them (for example, problems of fidelity), as well as the difficulties of a lonely existence (Rean, 2009);
  - dissatisfaction with their marital status. For example, it has been established that there are significantly fewer happy men and women among singles and in a civil marriage than among those who are married. Although, of course, cohabitation partially relieves the feeling of loneliness, it does not always bring a feeling of happiness (Sinelnikov, 2018).

In general, our research has shown that, despite challenges, families tend to cope with them using exactly those resilience resources that best help the family to respond to a particular challenge. This is supported by Muriel Lin’s research (Lin et al., 2016). However, Lin et al. used a different model for assessing family resilience, and the results of the two studies are not completely comparable. Thus, the economic strength of the family, which turned out to be associated with confidence in overcoming crises related to health problems, was not assessed by the Family Resilience Scale. Family cohesion is highly correlated with participants’ confidence in resolving intrafamilial conflicts, while our results showed the importance of a positive outlook and confidence in solving problems as a useful response to relationship challenges. At the same time, both studies confirmed the importance of the ability to discuss problems together.

Flexibility in responding to and accepting the unexpectedness of life events helps with loss challenges (an event that cannot be changed). Family spirituality helps to cope with global challenges and challenges of illness. The ability to solve problems together becomes the leading resource in solving financial problems. A positive outlook and confidence in solving problems are effective responses to relationship challenges. This does not contradict the functional-dynamic scheme for the formation of family resilience developed by A.V. Makhnach, but only empirically confirms it. Th at author noted: “Each newly emerging need in the family will trigger a mechanism for restructuring the available internal and external resources that are adequate to the situation, providing one or another process (coping, adaptation, etc.) that allows it to achieve a certain goal at a specific period throughout its life cycle. If a different need arises, the same process will occur again, but the resources attracted by the family may be different, used in a different combination, in a different sequence.” (Makhnach, 2016, p.162).

The results of our research also showed the importance of family ties and family cohesion as resilience factors. Thus, family bonding remains the same when the family’s financial well-being is challenged, which is confirmed by the research by D.K. Orthner et al. (Orthner et al., 2004). Th at work described the potential sources of strength for low-income American families, one of which is cohesion. The connection becomes closer after challenges of illness, loss challenges, and global challenges. For example, recent research on family resilience in the face of loss shows that family cohesion is a key resource for family resilience (Khatib, 2022).

This is also evidenced by how the subscales of family resilience correlated with changes in family ties, and the answers of the respondents to the question of what helped their families survive the adverse event. It is illustrative that the most common responses were the characteristics of the family as a cohesive system. It is reasonable to say that the personal qualities listed by the respondents in response to this question are precisely those that allow an individual, as a member of a microsocial group, to grow as a person through overcoming difficulties and creating the preconditions for such growth of other family members. In turn, self-sacrifice as avoiding and building internal obstacles to meet communication needs and to openly discussing problems turned out to be the least mentioned. Overall, it can be said that close relationships in the family as a system, and the resources for personal and spiritual growth of its members, are what helps Russian families to cope with modern challenges.

Our results are significant for family counseling practice. Information about modern challenges and Russian families’ responses to them helps in developing consultative strategies aimed at updating and strengthening family resilience resources.

## Conclusion

1. Theoretical analysis of the problem showed that most researchers consider a challenge to be a psychological task that includes various events and the requirement to grow in order to solve the problem and give a timely adequate response; the challenge is seen as a “wake-up call” for family resilience. Our research confirmed this thesis. It has been found that the intensity of events becomes a signal for awakening family communication and connectedness, acceptance and flexibility, and family resilience as a whole.
2. The challenges for modern Russian families are loss challenges, relationship challenges, global challenges, challenges of illness, and the challenge of financial wellbeing. The highest intensity scores were given to global challenges, loss challenges, and challenges of illness. At the same time, the types of the indicated challenges weren’t related to such biographical facts as having children, disability, or certain type of occupation, but were related to marital status (challenges of illness were mentioned more often by married people; relationship challenges by unmarried/single persons and those in unregistered marriage; and global challenges by divorced and widowed people) and to age (the respondents who wrote in global challenges and challenges of illness are on average about 40 years old; those who survived loss and relationship challenges were on average of just over 30 years old; and those who indicated events associated with serious financial difficulties were about 35 years old).
3. Different types of challenges affected family relationships in different ways: family relationships remained as close as they were previously when the financial well-being of the family was threatened; family members were more likely to move away from each other when their relationships were challenged; family members became closer to each other in the face of illnesses, loss, and global challenges.
4. Family resilience turned out to be an adequate response to modern challenges. Loss challenges stimulate flexibility of response, acceptance, and overcoming suffering through mutual understanding. Global challenges and challenges of illness awaken family spirituality (common values, meanings, and moral principles). The resources which help families overcome challenges of financial well-being are things like the ability to discuss problems together and to share decision making.

## Limitations

Our sample was not balanced by gender, since the study was voluntary, and women were more willing to participate. Therefore, it is necessary to continue the research with a larger and more balanced sample. The duration of the experienced adverse events was not taken into account, which could also affect the evaluation of their intensity. At the same time, the number of participants was sufficient for a pilot study, considering the life context of Russian families and dealing with nomothetic and idiographic approaches. All these factors ensure the reliability of our data and determine the prospects for further research.
